# COPD exacerbations and patient-reported outcomes according to post-bronchodilator FEV_1_ – a post-hoc analysis of pooled data

**DOI:** 10.1186/s12890-023-02436-1

**Published:** 2023-04-28

**Authors:** Chee-Shee Chai, Diana-Leh-Ching Ng, Sumastika Bt Mos, Muhammad Amin B Ibrahim, Seng-Beng Tan, Yong-Kek Pang, Chong-Kin Liam

**Affiliations:** 1 Department of Medicine, Faculty of Medicine and Health Science, University Malaysia Sarawak, Kota Samarahan, Sarawak, Malaysia; 2 Department of Nursing, Faculty of Medicine and Health Science, University Malaysia Sarawak, Kota Samarahan, Sarawak, Malaysia; 3 Department of Medicine, Faculty of Medicine, University Technology MARA, Sungai Buloh, Selangor Malaysia; 4 Department of Medicine, Faculty of Medicine, University of Malaya, Kuala Lumpur, Malaysia

**Keywords:** Chronic obstructive pulmonary disease (COPD), Forced expiratory volume in one second (FEV_1_), Exacerbations, Modified Medical Research Council (mMRC), COPD Assessment Test (CAT), St George’s respiratory questionnaire for COPD (SGRQ-c)

## Abstract

**Background:**

Management strategies of chronic obstructive pulmonary disease (COPD) need to be tailored to the forced expiratory volume in one second (FEV_1)_, exacerbations, and patient-reported outcomes (PROs) of individual patients. In this study, we analyzed the association and correlation between the FEV_1_, exacerbations, and PROs of patients with stable COPD.

**Methods:**

This was a post-hoc analysis of pooled data from two cross-sectional studies that were previously conducted in Malaysia from 2017 to 2019, the results of which had been published separately. The parameters measured included post-bronchodilator FEV_1_ (PB-FEV_1_), exacerbations, and scores of modified Medical Research Council (mMRC), COPD Assessment Test (CAT), and St George’s Respiratory Questionnaire for COPD (SGRQ-c). Descriptive, association, and correlation statistics were used.

**Results:**

Three hundred seventy-four patients were included in the analysis. The PB-FEV_1_ predicted was < 30% in 85 (22.7%), 30–49% in 142 (38.0%), 50–79% in 111 (29.7%), and ≥ 80% in 36 (9.6%) patients. Patients with PB-FEV_1_ < 30% predicted had significantly more COPD exacerbations than those with PB-FEV_1_ 30–49% predicted (p < 0.001), 50–79% predicted (p < 0.001), and ≥ 80% predicted (p = 0.002). The scores of mMRC, CAT, and SGRQ-c were not significantly higher in patients with more severe airflow limitation based on PB-FEV_1_ (p = 0.121–0.271). The PB-FEV_1_ predicted had significant weak negative correlations with exacerbations (r = − 0.182, p < 0.001), mMRC (r = − 0.121, p = 0.020), and SGRQ-c scores (r = − 0.114, p = 0.028). There was a moderate positive correlation between COPD exacerbations and scores of mMRC, CAT, and SGRQ-c (r = 0.407–0.482, all p < 0.001). There were significant strong positive correlations between mMRC score with CAT (r = 0.727) and SGRQ-c scores (r = 0.847), and CAT score with SGRQ-c score (r = 0.851) (all p < 0.001).

**Conclusions:**

In COPD patients, different severity of airflow limitation was not associated with significant differences in the mMRC, CAT, and SGRQ-c scores. Exacerbations were significantly more frequent in patients with very severe airflow limitation only. The correlation between airflow limitation with exacerbations, mMRC, and SGRQ-c was weak.

**Supplementary Information:**

The online version contains supplementary material available at 10.1186/s12890-023-02436-1.

## Background

“Chronic obstructive pulmonary disease (COPD) is a common, preventable, and treatable airway disease characterized by persistent respiratory symptoms and airflow limitation caused by prolonged exposure to noxious particles or gases.” [1] The estimated global prevalence of COPD is 13.1% with a continent-based prevalence of 12.4% in Europe, 13.2% in the Americas, 13.5% in Asia, 11.6% in Oceania, and 13.9% in Africa [2]. Worldwide, COPD is currently the fourth leading cause of mortality resulting in 3.2 million deaths annually, and the second leading cause of disease burden accounting for 63.9 million disability-adjusted life-years [3].

Forced expiratory volume in one second (FEV_1_), exacerbations, and patient-reported outcomes (PROs) such as dyspnea symptom and health-related quality of life (HRQOL) are important endpoints used to evaluate the severity of COPD and its impacts on health [4] COPD patients with lower FEV_1_ have been shown to have a significantly higher exacerbation frequency, symptoms burden, disabilities, and all-causes mortalities [5]. Similarly, those who experience frequent COPD exacerbations are shown to have poorer lung function, exercise tolerance, and survival [6, 7]. Physical disabilities, sleep disturbances, psychological distress, and comorbidities are more common in COPD patients who report higher symptoms burden or poorer HRQOL [8, 9]. Besides, these patients also have a poorer long-term prognosis, such as being at higher risk of exacerbations, hospitalizations, and death [8, 9].

COPD is a heterogenous disease, of which its management strategies need to be tailored to the FEV_1_, exacerbations, and PROs of individual patients [10]. However, data on the relationship between these clinical endpoints in COPD patients are generally limited to the Western population and in clinical trials setting [11]. Studies have shown COPD patients in Asia have different clinical phenotypes, [12] as well as higher disability, hospitalisations, and mortality compared to those in advanced Western countries [13]. Besides, poverty, pulmonary tuberculosis, and indoor biomass fuel burning are unique and yet common risks of COPD in Asia. [14, 15] Difference in cultural background, literacy level, languages, and daily activities between Asia and Western population may cause discrepancy in COPD PROs response [16]. Compared to in real-world, patients involved in COPD clinical trials often report better clinical endpoints due to various advantages such as sample selection, use of newer drugs, and Hawthrone effect. [17, 18] Therefore, it is essential to look into these clinical endpoints among patients in Asia and real-world setting in order to develop a region-specific evidence-based management policy for COPD .

In this study, we analysed the relationship between FEV_1_, exacerbations, and PROs among patients with stable COPD in Malaysia. The primary objective was to determine the association between the objectively measured FEV_1_ versus subjectively reported exacerbations and PROs. The secondary objective was to determine the correlation and its strength between FEV_1_, exacerbations, and PROs.

## Methods

### Study Design and patients

This is a post-hoc analysis of pooled data from two cross-sectional studies that were previously conducted in Malaysia from 2017 to 2019, the results of which had been published separately. [19, 20] Both studies aimed to compare the HRQOL of patients with stable COPD according to their clinical phenotypes. The inclusion criterion of the first study was patients aged 40 years and above with the ratio of post-bronchodilator FEV_1_ (PB-FEV_1_) to post-bronchodilator forced vital capacity (PB-FVC) of < 0.7; while the inclusion criterion for the second study was patients aged 35 years and above with the ratio of PB-FEV_1_ to PB-FVC in six seconds (PB-FVC_6_) of < 0.7. Otherwise, both studies had similar exclusion criteria as reported. These studies were conducted in accordance with the Declaration of Helsinki. Ethics approval was obtained from the respective institutions and all patients provided written informed consent.

### Procedure

In both studies, eligible patients were consecutively identified from the respective clinics. Spirometry was performed according to the American Thoracic Society and European Respiratory Society guidelines [21]. Patient demographic characteristics and exacerbation frequency were obtained by face-to-face interviews and from the case notes. Demographic characteristics included age, gender, ethnicity, smoking status, smoking quantity, and history of biomass smoke exposure. The PROs included the modified Medical Research Council (mMRC) Dyspnea Scale, COPD Assessment Test (CAT), and St. George Respiratory Questionnaire for COPD (SGRQ-c) which patients were instructed to answer independently. (Supplement 1).

PB-FEV_1_ was expressed in percent of predicted value based on the patients’ age, gender, ethnicity, and height. Patients were divided into four groups according to the severity of airflow limitation based on PB-FEV_1_: < 30% predicted (very severe); 30–49% predicted (severe); 50–79% predicted (moderate); and ≥ 80% predicted (mild) [1]. The ethnic composition of Malaysians includes Malay, Chinese, Indian, and others (such as natives). A never smoker was defined as an individual who smoked < 100 cigarettes in a lifetime; while former or current smokers were individuals who had smoked ≥ 100 cigarettes in a lifetime [22]. An former smoker was one who had quit smoking for more than a year at the time of interview. Smoking quantity was calculated in pack-year (number of cigarettes smoked per day/20 x number of years smoking). Exposure to biomass smoke was defined as ever exposure to smoke from the burning of wood, crop, or charcoal for ≥ 100 h per year [23]..

Both studies only counted the number of moderate and severe exacerbations over the past one year that constitutes the definition of “exacerbator” [10]. A moderate exacerbation was defined as acute worsening of COPD symptoms that required outpatient treatment with corticosteroids and/or antibiotics; whereas a severe exacerbation was the one that warranted hospitalization [24]. Patients’ mMRC, [25] CAT, [26] and SGRQ-c scores were calculated and interpreted as per original validation of the questionnaires. [27, 28].

### Statistical analysis

Categorical variables were expressed as percentages while continuous variables were expressed as mean ± standard deviation (SD) or median with interquartile range. The difference between groups for the categorical variables was compared using the chi-squared test, with post-hoc analysis taking adjusted standardized residual of > 2 as significant. The difference between more than two groups for the continuous variables was compared using the one-way ANOVA test or Kruskal-Wallis H test. The post-hoc analysis for the former was the Tukey test and for the latter was Dunn’s procedure with Bonferroni adjustment. The correlation of continuous variables was calculated using Pearson or Spearman correlation test followed by linear regression test. The correlation coefficient (r) was defined as weak or small (0.10–0.29); moderate or medium (0.30–0.49); and strong or large (≥ 0.50) based on Cohen classification [29]. A 2-sided p-value of less than 0.05 was considered significant. Statistical analyses were performed using Statistical Package for the Social Sciences (SPSS for Windows version 25.0, SPSS Inc, Chicago, IL, USA).

## Results

### Demographic characteristics

A total of 374 patients were included in the analysis (Fig. 1). Their demographic characteristics are shown in Table 1. The overall mean PB-FEV_1_ was 47.5 ± 21.3% predicted. The PB-FEV_1_ was < 30% predicted in 85 patients (22.7%), 30–49% predicted in 142 patients (38.0%), 50–79% predicted in 111 patients (29.7%), and ≥ 80% predicted in 36 patients (9.6%). The proportion of patients with a history of biomass smoke exposure was significantly higher in those with PB-FEV_1_ < 30% predicted (48.2%) compared to those with less severe airflow limitation [PB-FEV_1_ 30–49% (31.7%), PB-FEV_1_ 50–79% (35.1%), and PB-FEV_1_ ≥ 80% (19.4%), p = 0.012]. Otherwise, the age, gender, ethnicity, smoking status, and smoking quantity of the patients were not significantly different across the different categories of severity of airflow limitation based on PB-FEV_1_.

**Fig. 1:**

Algorithm of patients’ recruitment in the study

**Table 1:** Demographic characteristics of COPD patients according to the severity of airflow limitation based on PB-FEV_1_

Characteristics	All COPD patients, N = 374	Severity of airflow limitation based on PB-FEV_1_ (% predicted)				p-value
		< 30%	30–49%	50–79%	≥ 80%	
		Number of COPD patients (% of total COPD patients)				
		85 (22.7)	142 (38.0)	111 (29.7)	36 (9.6)	
Age, years (mean ± SD, 95% CI)	67.3 ± 11.8; 66.1–68.5	65.7 ± 10.5; 63.4–67.9	67.7 ± 11.4; 65.8–69.6	67.2 ± 12.4; 64.9–69.5	70.0 ± 13.7; 65.1–74.4	0.345
Gender, n (%) Male Female	317 (84.8) 57 (15.2)	77 (90.6) 8 (9.4)	120 (84.5) 22 (15.5)	90 (81.1) 21 (18.9)	30 (83.3) 6 (16.7)	0.326
Ethnicity, n (%) Malay Chinese Indian Others	104 (27.8) 104 (27.8) 31 (8.3) 135 (36.1)	24 (28.2) 18 (21.2) 6 (7.1) 37 (43.5)	42 (29.6) 37 (26.1) 14 (9.9) 49 (34.5)	27 (24.3) 33 (29.7) 11 (9.9) 40 (36.0)	11 (30.6) 16 (44.4) 0 9 (25.0)	0.283
Smoking status, n (%) Never smoker Former smoker Current smoker	69 (18.4) 201 (53.7) 104 (27.8)	13 (15.3) 45 (52.9) 27 (31.8)	27 (19.0) 78 (54.9) 37 (26.1)	22 (19.8) 54 (48.6) 35 (31.5)	7 (19.4) 24 (66.7) 5 (13.9)	0.422
Smoking quantity, pack-years (mean ± SD, 95% CI)	30.2 ± 27.6; 27.4–33.0	27.1 ± 22.3; 22.3–31.9	30.5 ± 29.5; 25.6–35.3	32.6 ± 29.3; 27.1–38.1	28.4 ± 25.6; 19.8–37.1	0.559
Biomass smoke exposure, n (%) No Yes	242 (64.7) 132 (35.3)	44 (51.8) 41 (48.2)	97 (68.3) 45 (31.7)	72 (64.9) 39 (35.1)	29 (80.6) 7 (19.4)	0.012

Abbreviations: COPD, chronic obstructive pulmonary disease; PB-FEV_1_, post-bronchodilator forced expiratory volume in one second;

SD, standard deviation; CI, confidence interval

### PB-FEV_1_ and COPD exacerbations

The frequency of all and moderate COPD exacerbations was significantly different across the patient groups with different severity of airflow limitation based on PB-FEV_1_ (Table 2). Patients with PB-FEV_1_ < 30% predicted had significantly more frequent exacerbations of all types than those with PB-FEV_1_ 30–49% predicted (4.0 versus 1.8, p < 0.001), PB-FEV_1_ 50–79% predicted (4.0 versus 1.6, p < 0.001), and PB-FEV_1_ ≥ 80% predicted (4.0 versus 1.1, p = 0.002). Moderate exacerbations were also significantly more frequent in patients with PB-FEV_1_ < 30% predicted than that of patients with PB-FEV_1_ 30–49% predicted (3.0 versus 1.0, p < 0.001), PB-FEV_1_ 50–79% predicted (3.0 versus 1.0, p < 0.001) and PB-FEV_1_ ≥ 80% predicted (3.0 versus 0.7, p = 0.001). When compared between the COPD patients with PB-FEV_1_ 30–49% predicted, PB-FEV_1_ 50–79% predicted, and PB-FEV_1_ ≥ 80% predicted, there was no significant difference in the frequency of all (p = 0.851–0.984) and moderate exacerbation (p = 0.970–0.999). Even though patients with more severe airflow limitation had more frequent severe exacerbations, the difference was not significant (p = 0.106).

**Table 2:** Exacerbation frequency of COPD patients according to the severity of airflow limitation based on PB-FEV_1_

Exacerbation frequency	All COPD patients, N = 374	Severity of airflow limitation based on PB-FEV_1_ (% predicted)				p-value
		< 30%	30–49%	50–79%	≥ 80%	
		Number of COPD patients (% of total COPD patients)				
		85 (22.7)	142 (38.0)	111 (29.7)	36 (9.6)	
Total, episodes (mean ± SD, 95% CI) Moderate, episodes (mean ± SD, 95% CI) Severe, episodes (mean ± SD, 95% CI)	2.2 ± 4.2; 1.7–2.6 1.4 ± 3.1; 1.1–1.7 0.8 ± 1.5; 0.6–0.9	4.0 ± 7.0; 2.5–5.5 3.0 ± 5.5; 1.8–4.2 1.1 ± 2.0; 0.6–1.5	1.8 ± 2.9; 1.3–2.2 1.0 ± 1.8; 0.7–1.3 0.8 ± 1.4; 0.6–1.0	1.6 ± 2.7; 1.1–2.1 1.0 ± 1.8; 0.6–1.3 0.6 ± 1.3; 0.4–0.9	1.1 ± 1.8; 0.5–1.8 0.7 ± 1.1; 0.3–1.1 0.5 ± 0.8; 0.2–0.8	< 0.001 < 0.001 0.106

Abbreviations: COPD, chronic obstructive pulmonary disease; PB-FEV_1_, post-bronchodilator forced expiratory volume in one second;

SD, standard deviation; CI, confidence interval

### PB-FEV_1_ and PROs – mMRC, CAT, and SGRQ-c

The patients’ CAT and SGRQ-c scores were higher than normal regardless of their PB-FEV_1_ (Table 3). Patients with more severe airflow limitation based on their PB-FEV_1_ had higher scores of mMRC, CAT (total) and SGRQ-c (total) compared to those with less severe airflow limitation, but the difference was not statistically significant (p = 0.053, p = 0.230, and p = 0.121, respectively).

**Table 3:** PROs of COPD patients according to the severity of airflow limitation based on PB-FEV_1_

PROs	All COPD patients, N = 374	Severity of airflow limitation based on PB-FEV_1_ (% predicted)				p-value
		< 30%	30–49%	50–79%	≥ 80%	
		Number of COPD patients (% of total COPD patients)				
		85 (22.7)	142 (38.0)	111 (29.7)	36 (9.6)	
mMRC, score (mean ± SD, 95% CI)	1.7 ± 1.3; 1.6–1.9	2.0 ± 1.4; 1.7–2.3	1.8 ± 1.3; 1.6–2.1	1.6 ± 1.1; 1.4–1.8	1.4 ± 1.1; 1.1–1.8	0.053
CAT, total score (mean ± SD, 95% CI)	14.8 ± 10.1; 13.8–15.8	15.7 ± 11.1; 13.3–18.1	14.9 ± 10.1; 13.2–16.5	15.1 ± 9.9; 13.2–17.0	11.6 ± 8.1; 8.9–14.4	0.230
SGRQ-c, % (mean ± SD, 95% CI) Total Symptoms Activities Impact	40.4 ± 26.0; 37.8–43.1 42.7 ± 26.4; 40.0–45.3 48.6 ± 30.4; 45.5–51.7 34.8 ± 29.4; 31.8–37.8	43.6 ± 29.2; 37.3–49.9 47.0 ± 29.2; 40.7–53.3 53.8 ± 32.5; 46.8–60.8 36.5 ± 32.9; 29.4–43.6	41.3 ± 27.1; 36.8–45.8 44.1 ± 27.8; 39.5–48.8 50.4 ± 32.1; 45.0–55.7 34.9 ± 29.2; 30.1–39.8	39.8 ± 23.7; 35.4–44.3 41.1 ± 22.7; 36.8–45.4 44.5 ± 27.2; 39.4–49.7 36.6 ± 28.8; 31.2–42.0	31.4 ± 18.5; 25.1–37.7 31.8 ± 21.4; 24.6–39.1 42.2 ± 26.1; 33.3–51.0 25.0 ± 21.5; 17.7–32.2	0.121 0.026 0.087 0.192

Abbreviations: PROs, patient-reported outcomes; COPD, chronic obstructive pulmonary disease; PB-FEV_1_, post-bronchodilator forced expiratory volume in one second; mMRC, modified Medical Research Council; COPD Assessment Tool; SGRQ-c, St George’s Respiratory Questionnaire for COPD; SD, standard deviation; CI, confidence interval

There was no significant difference in the scores of each of the SGRQ-c domains according to the severity of airflow limitation based on PB-FEV_1_, except for symptoms. For the symptoms domain, patients with PB-FEV_1_ < 30% predicted had a significantly higher score than those with PB-FEV_1_ ≥ 80% predicted (47.0 versus 31.8, p = 0.020). The score of symptoms domain, however, was not statistically different between COPD patients with PB-FEV_1_ < 30% predicted, PB-FEV_1_ 30–49% predicted, and PB-FEV_1_ 50–79% predicted (p = 0.405–0.858); or between those with PB-FEV_1_ 30–49% predicted, PB-FEV_1_ 50–79% predicted, and PB-FEV_1_ ≥ 80% predicted (p = 0.058–0.798).

### Correlation and regression between PB-FEV_1_, exacerbations, and PROs

There were weak negative correlations between PB-FEV_1_ and exacerbations (r = − 0.182, p < 0.001), mMRC score (r = − 0.121, p = 0.020), and SGRQ-c score (r = − 0.114, p = 0.028), respectively (Table 4). There was no correlation between PB-FEV_1_ and CAT score (r = − 0.072, p = 0.162). Exacerbations were positively correlated to the mMRC, CAT, and SGRQ-c scores, respectively with moderate co-efficient (r = 0.407–0.482, all p < 0.001). There were strong positive correlations between mMRC score and CAT score (r = 0.727) and SGRQ-c score (r = 0.847), respectively while CAT score was strongly correlated to SGRQ-c score (r = 0.851) (all p < 0.001). The linear regressions involving PB-FEV_1_, exacerbations, mMRC, CAT, and SGRQ-c are as shown in Table 4.

**Table 4:** Pearson correlation and linear regression between PB-FEV_1,_ exacerbations, and PROs of COPD patients

Variables	R*	*R* ^*2*^ *	Constant*	B*	SE*	r^#^/beta*	t*	p-value^
PB-FEV_1_% – Exacerbations mMRC CAT SGRQ-c	0.183 0.121 0.072 0.114	0.033 0.015 0.005 0.013	3.870 2.085 16.439 47.049	− 0.036 − 0.007 − 0.035 − 0.139	0.010 0.003 0.025 0.063	− 0.183 − 0.121 − 0.072 − 0.114	-3.588 -2.344 -1.401 -2.204	< 0.001 0.020 0.162 0.028
Exacerbations – mMRC CAT SGRQ-c	0.407 0.445 0.482	0.165 0.198 0.233	1.478 12.480 33.968	0.123 1.075 2.999	0.014 0.112 0.282	0.407 0.445 0.482	8.587 9.573 10.619	< 0.001 < 0.001 < 0.001
mMRC – CAT SGRQ-c	0.727 0.847	0.529 0.717	4.656 10.062	5.819 17.425	0.285 0.568	0.727 0.847	20.436 30.676	< 0.001 < 0.001
CAT – SGRQ-c	0.851	0.724	8.039	2.189	0.070	0.851	31.251	< 0.001

* value for linear regression

# value for Pearson correlation

^ value for both Pearson correlation and linear regression

Abbreviations: PB-FEV_1_, post-bronchodilator forced expiratory volume in one second; PROs, patient-reported outcomes; COPD, chronic obstructive pulmonary disease; mMRC, modified Medical Research Council; CAT, COPD Assessment Tool; SGRQ-c, St George’s Respiratory Questionnaire for COPD; B, unstandardized beta; SE, standard error

## Discussion

The majority (60.7%) of the COPD patients in this analysis had severe or very severe airflow limitation. Biomass smoke exposure was significantly more common among patients with very severe airflow limitation. Similarly, all and moderate COPD exacerbations were significantly more frequent in those with very severe airflow limitation. Severe exacerbations, mMRC, CAT and SGRQ-c scores, however, were not significantly different across the severity of airflow limitation groups. PB-FEV_1_ was only weakly correlated with exacerbations, mMRC, and SGRQ-c; exacerbations were moderately correlated with mMRC, CAT, and SGRQ-c; while mMRC was strongly correlated with CAT and SGRQ-c.

Hurst el at reported COPD exacerbations were more frequent and more severe as the severity of airflow limitation increased [24]. Oca et al. and Lutter et al. respectively reported significantly more frequent exacerbations among patients with severe to very severe airflow limitation. [30, 31] A study by Bikov et al. reported COPD patients in the lowest quantiles (FEV_1_ < 53.5% predicted) of airflow limitation had a significantly higher risk of moderate-to-severe and severe exacerbations [32]. Halpin et al. and a systemic review by Westwood et al. reported an inverse relationship between exacerbations and FEV_1_ but the correlation was weak (r = − 0.12, and − 0.27, respectively). [33, 34] The findings of significantly more frequent exacerbations in our patients with very severe airflow limitation and a weak correlation with PB-FEV_1_ are consistent with the findings of these studies.

Even though studies have shown COPD patients with more severe airflow limitation reported higher mMRC score, the significant of this association was not reported [35]. With regard to FEV_1_ and mMRC, Oga et al. reported a moderate correlation (r = − 0.37), [36] whereas Huang et al. and Kostikas et al. reported a weak correlation (r = − 0.234 and − 0.121, respectively). [37, 38] ) The present analysis showed a weak negative correlation between PB-FEV_1_ and mMRC is similar to that reported by Huang et al. and Kostikas et al. [37, 38].

Unlike the present study, Jones et al. and Ghobadi et al. reported significantly higher CAT score, [39, 40]; while Agrawal et al. and Balcells et al. reported significantly higher SGRQ-c score in COPD patients with more severe airflow limitation. [41, 42] Majority of the existing studies reported a moderate-to-strong negative correlation between FEV_1_ and SGRQ-c (r = − 0.372 to − 0.86). [34, 36, 43–45] Only Sundh et al. reported a weak negative correlation between FEV_1_ and CAT ( r = − 0.13) that similar to our study [46]..

Studies have shown patients with frequent COPD exacerbations have significantly higher mMRC, CAT, and SGRQ-c scores. . [47, 48] For exacerbations, a moderate positive correlation with mMRC (r = 0.31) and CAT (r = 0.42) was reported by Kelly et al., [49] while with SGRQ-c was reported by Burgel et al. (r = 0.31) and Deslee et al. (r = 0.391), respectively. [44, 50] A strong positive correlation between mMRC, CAT, and SGRQ-c (r = 0.50–0.88) was consistently reported in numerous studies of COPD. [43, 44, 49–51] In our study, similar correlation and strength was also seen between exacerbations and PROs, as well as between the PROs.

The present study highlighted that there was a lack of association and only a weak correlation between FEV_1,_ mMRC, and SGRQ-c in patients with COPD in Malaysia when compared to studies in other part of the world. Environment, genetic, and cultural factors that cause different in clinical characteristics and PROs response of these patients could be the main explanation. [12, 14–16] FEV_1_ is a unidimensional measurement that reflects the pathophysiology of COPD while exacerbations and PROs are multidimensional measurements of COPD consequences from the patients’ perspective [40]. This explains the absence or small correlation between FEV_1_ with exacerbations and PROs in this study. The interaction between smoking, air pollution, respiratory tract infection, bronchiectasis, blood eosinophil count, the severity of airflow limitation, prior exacerbations, and comorbidities leads to the occurrence of COPD exacerbation [52]. For dyspnea symptom, the mechanisms encompass the interaction of physiological, psychological, and emotional factors of COPD patients [53]. Meanwhile, the HRQOL of COPD patients depends on the interaction of their physical, functional, emotional, social, and economic well-being [54]. The moderate correlation between COPD exacerbations, mMRC, CAT, and SGRQ-c in this study could be attributed to the partial overlap between their dimensions. On the other hand, most of the dimensions are overlapped between mMRC, CAT, and SGRQ-c explains the strong correlation. Even though symptoms such as cough, sputum, and wheezing are also assessed, the majority of questions in CAT (CAT 3 – CAT 8) and SQRQ-c (part of symptoms component, most of activity and impact component) still assess dyspnea symptom or their complications as the main outcomes. [26, 27].

The findings from this study suggest that FEV_1_ is not a reliable parameter to measure during the follow-up of COPD patients in Malaysia due to its weak correlation with exacerbations and other PROs. Exacerbations should be routinely assessed as it is the prognosis hallmark of COPD and is only moderately reflected by the mMRC, CAT, or SGRQ-c. mMRC can predicts the value of CAT and SGRQ-c strongly. Therefore, during a busy clinic, a simpler and time-saving tool such as mMRC should precede CAT and SGRQ-c. In short, exacerbations and mMRC are still the recommended parameters to evaluate during the follow-up of COPD patients in Malaysia. This is in line with the Global Initiative for Chronic Obstructive Lung Disease guidelines that recommend grouping of COPD patients based on exacerbation frequency in the past one year and mMRC or CAT score at diagnosis and evaluating exacerbations and dyspnea symptom during follow-up visits [1]. Only in selected circumstances, spirometry is used to identify alternative diagnoses, suitability for interventional procedures, and to detect a rapid decline in FEV_1_.

This study evaluates the core endpoints of COPD simultaneously, namely FEV_1_, exacerbations, mMRC, CAT, and SGRQ-c. Even though studies looking at these endpoints have been conducted in other parts of the world, this is one of the very few studies in the South-East Asia region. Data from different regions of the world is needed for various reasons. First, the etiology of COPD can be different. In the present study, biomass smoke exposure was reported in more than one-third of the patients. Even though FEV_1_ decline due to biomass smoke exposure is slower, [55] biomass smoke exposure and cigarette smoking have an additive adverse effect on airflow obstruction [56]. Second, genetic heterogeneity can affect the presentation and outcomes of COPD. This study included the population from Peninsular Malaysia and the Island of Borneo. Third, the culture and economic activity of the population could have an impact on the perceived symptom and HRQOL. Other strengths of this study include representative sample was obtained from both the primary and tertiary care centers, as well as a similar methodology was used in both studies, therefore, minimizing any data bias. However, there are several limitations to this study. First, this was a cross-sectional study. PROs may vary over time and such variation may not be reflected in a cross-sectional study. Serial changes of FEV_1_ may give a better understanding of the exacerbations and PROs. Second, the inclusion criteria of the studies were slightly different in terms of age and definition of fixed airway obstruction. The use of PB-FVC_6_ in the second study potentially excludes a proportion of patients with mild COPD. Third, the study outcome was decided later in this post-hoc analysis. However, this study fulfilled the minimum sample size of 208 patients as calculated. Fourth, recall errors in exacerbation frequency cannot be discounted but minimized by counter checks with the medical records and family members. A future study that prospectively evaluates the FEV_1_, exacerbations, and PROs among COPD patients in local setting is expected to mitigate these limitations.

## Conclusions

We conclude that different severity of airflow limitation based on PB-FEV_1_ was not associated with significant differences in the mMRC, CAT, or SGRQ-c of COPD patients. Exacerbations was significantly more frequent in patients with very severe airflow limitation only. The correlation between the severity of airflow limitation with exacerbations, mMRC, CAT, and SGRQ-c was weak. Therefore, PB-FEV_1_ measurement during the routine clinic follow-up of COPD patients does not provide additional information on top of mMRC or CAT scores and exacerbations history that may influence treatment decisions.

## Electronic supplementary material

Below is the link to the electronic supplementary material.

Supplementary Material 1

## Data Availability

The datasets used and/or analyzed during the current study are available from the corresponding author on reasonable request.

## Abbreviations

COPD: chronic obstructive pulmonary disease
FEV1: forced expiratory volume in one second
PROs: patient-reported outcomes
HRQOL: health-related quality of life
PB-FEV1: post-bronchodilator FEV_1_
PB-FVC: post-bronchodilator forced vital capacity
PB-FVC6: PB-FVC in six seconds
mMRC: modified Medical Research Council
CAT: COPD Assessment Tool
HRQOL: health-related quality of life
SGRQ-c: St George’s Respiratory Questionnaire for COPD
SD: standard deviation
r: correlation coefficient
CI: confidence interval
B: unstandardized beta
SE: standard error
