# Supplementary material for: COPD exacerbations and patient-reported outcomes according to post-bronchodilator FEV1 – a post-hoc analysis of pooled data
Source: BMC Pulm Med. 2023 Apr 28;23:150. doi: 10.1186/s12890-023-02436-1 (PMC10148499; doi:10.1186/s12890-023-02436-1)
Supplement: Supplementary file 1 — Supplementary Material 1 [file 12890_2023_2436_MOESM1_ESM.pdf]

Modified Medical Research Council Dyspnoea Scale

0	"I only get breathless with strenuous exercise"
1	"I get short of breath when hurrying on the level or walking up a slight hill"
2	"I walk slower than people of the same age on the level because of breathlessness or have to stop for breath when walking at my own pace on the level"
3	"I stop for breath after walking about 100 yards or after a few minutes on the level"
4	"I am too breathless to leave the house" or "I am breathless when dressing"

Doherty DE et al. COPD: Consensus Recommendations for early diagnosis and treatment. Journal of Family Practice, Nov 2006

Your name:______________________

Today's date:______________________

How is your COPD? Take the COPD Assessment Test™ (CAT)

This questionnaire will help you and your healthcare professional to measure the impact that COPD (Chronic Obstructive Pulmonary Disease) is having on your wellbeing and daily life. Your answers and test score can be used by you and your healthcare professional to help improve the management of your COPD and gain the greatest benefit from the treatment.

For each item below, place a mark (X) in the box that best describes your current situation. Please ensure that you only select one response for each question.

Example: I am very happy [0 | X 1 | 2 | 3 | 4 | 5] I am very sad

	0	1	2	3	4	5		SCORE
I never cough	☐	☐	☐	☐	☐	☐	I cough all the time	☐
I have no phlegm (mucus) on my chest at all	☐	☐	☐	☐	☐	☐	My chest is full of phlegm (mucus)	☐
My chest does not feel tight at all	☐	☐	☐	☐	☐	☐	My chest feels very tight	☐
When I walk up a hill or a flight of stairs I am not out of breath	☐	☐	☐	☐	☐	☐	When I walk up a hill or a flight of stairs I am completely out of breath	☐
I am not limited to doing any activities at home	☐	☐	☐	☐	☐	☐	I am completely limited to doing all activities at home	☐
I am confident leaving my home despite my lung condition	☐	☐	☐	☐	☐	☐	I am not confident leaving my home at all because of my lung condition	☐
I sleep soundly	☐	☐	☐	☐	☐	☐	I do not sleep soundly because of my lung condition	☐
I have lots of energy	☐	☐	☐	☐	☐	☐	I have no energy at all	☐

TOTAL SCORE ☐☐

A COPD assessment test was developed by an interdisciplinary group of international COPD experts with support from GSK. GSK's activities in connection with the COPD assessment test are monitored by a supervisory council that includes external, independent experts, one of which is chair of the council.

CAT, the COPD assessment test and the CAT logo are trademarks that belong to the GSK group of companies. ©2009 GSK. All rights reserved.

ST. GEORGE'S RESPIRATORY QUESTIONNAIRE
for COPD patients

(SGRQ-C)

This questionnaire is designed to help us learn much more about how your breathing is troubling you and how it affects your life.
We are using it to find out which aspects of your illness cause you most problems, rather than what the doctors and nurses think your problems are.

Please read the instructions carefully and ask if you do not understand anything. Do not spend too long deciding about your answers.

ID: ___________________________

Date: ______/________/________ (dd/mm/yy)

Before completing the rest of the questionnaire:

Please select one box to show how you describe your current health:

Very good	Good	Fair	Poor	Very poor
☐	☐	☐	☐	☐

Version: 1st Sept 2005

Copyright reserved
P.W. Jones, PhD FRCP
Professor of Respiratory Medicine,
St. George's University of London,
Cranmer Terrace
London SW17 ORE, UK.

Tel. +44 (0) 20 8725 5371
Fax +44 (0) 20 8725 5955

St. George's Respiratory Questionnaire
PART 1

Questions about how much chest trouble you have.

Please select **ONE** box for each question:

Question 1. I cough:

most days a week........................ ☐ a

several days a week .... ☐ b

only with chest infections........... ☐ c

not at all...................................... ☐ d

Question 2. I bring up phlegm (sputum):

most days a week........................ ☐ a

several days a week .... ☐ b

only with chest infections........... ☐ c

not at all...................................... ☐ d

Question 3. I have shortness of breath:

most days a week........................ ☐ a

several days a week .... ☐ b

not at all...................................... ☐ c

Question 4. I have attacks of wheezing:

most days a week........................ ☐ a

several days a week .... ☐ b

a few days a month..................... ☐ c

only with chest infections........... ☐ d

not at all...................................... ☐ e

continued…

Question 5. How many attacks of chest trouble did you have during the last year?

3 or more attacks .... ☐ a

1 or 2 attacks............................... ☐ b

none .... ☐ c

Question 6. How often do you have good days (with little chest trouble)?

no good days............................... ☐ a

a few good days .... ☐ b

most days are good .... ☐ c

every day is good........................ ☐ d

Question 7. If you have a wheeze, is it worse in the morning?

no .... ☐

yes... ☐

continued…

St. George's Respiratory Questionnaire
PART 2

8. ***How would you describe your chest condition?***

Please select ***ONE***:

Causes me a lot of problems or is the most important problem I have	☐	a
Causes me a few problems	☐	b
Causes no problem	☐	c

9. ***Questions about what activities usually make you feel breathless.***

For each statement please select ***the box*** that applies to you **these days**:

	True	False	
Getting washed or dressed...	☐	☐	a
Walking around the home...	☐	☐	b
Walking outside on the level..	☐	☐	c
Walking up a flight of stairs...	☐	☐	d
Walking up hills..	☐	☐	e

St. George's Respiratory Questionnaire
PART 2

10. ***Some more questions about your cough and breathlessness.***

For each statement please select ***the box*** that applies to you **these days**:

	True	False	
My cough hurts	☐	☐	a
My cough makes me tired	☐	☐	b
I am breathless when I talk..	☐	☐	c
I am breathless when I bend over	☐	☐	d
My cough or breathing disturbs my sleep..	☐	☐	e
I get exhausted easily..	☐	☐	f

11. ***Questions about other effects that your chest trouble may have on you.***

For each statement please select ***the box*** that applies to you **these days**:

	True	False	
My cough or breathing is embarrassing in public................................	☐	☐	a
My chest trouble is a nuisance to my family, friends or neighbours.....	☐	☐	b
I get afraid or panic when I cannot get my breath	☐	☐	c
I feel that I am not in control of my chest problem...............................	☐	☐	d
I have become frail or an invalid because of my chest.........................	☐	☐	e
Exercise is not safe for me	☐	☐	f
Everything seems too much of an effort...	☐	☐	g

continued…

St. George's Respiratory Questionnaire
PART 2

12. ***These are questions about how your activities might be affected by your breathing.***

For each statement please select ***the box*** that applies to you **because of your breathing**:

	True	False	
I take a long time to get washed or dressed..	☐	☐	a
I cannot take a bath or shower, or I take a long time	☐	☐	b
I walk slower than other people, or I stop for rests................................	☐	☐	c
Jobs such as housework take a long time, or I have to stop for rests....	☐	☐	d
If I walk up one flight of stairs, I have to go slowly or stop	☐	☐	e
If I hurry or walk fast, I have to stop or slow down	☐	☐	f
My breathing makes it difficult to do things such as walk up hills, carrying things up stairs, light gardening such as weeding, dance, play bowls or play golf	☐	☐	g
My breathing makes it difficult to do things such as carry heavy loads, dig the garden or shovel snow, jog or walk at 5 miles per hour, play tennis or swim..	☐	☐	h

13. ***We would like to know how your chest trouble usually affects your daily life.***

For each statement please select ***the box*** that applies to you **because of your breathing**:

	True	False	
I cannot play sports or games	☐	☐	a
I cannot go out for entertainment or recreation	☐	☐	b
I cannot go out of the house to do the shopping	☐	☐	c
I cannot do housework	☐	☐	d
I cannot move far from my bed or chair...	☐	☐	e

continued…

St. George's Respiratory Questionnaire

14. ***How does your chest trouble affect you?***

Please select ***ONE***:

It does not stop me doing anything I would like to do .... ☐ a

It stops me doing one or two things I would like to do................. ☐ b

It stops me doing most of the things I would like to do................ ☐ c

It stops me doing everything I would like to do............................ ☐ d

Thank you for filling in this questionnaire.

Before you finish, would you please check to see that you have answered all the questions.
